# A New Anæsthetic Kerosolene

**Published:** 1861-10

**Authors:** H. J. Bigelow

**Affiliations:** Boston


### Selections.

A New Anæsthetic—Kerosolene.—At the meeting of the Boston Society for Medical Improvement, held on Monday evening last, a liquid bearing the above name, and suspected of possessing anæsthetic properties, was presented by Dr. Bowditch, from Mr. Merrill, and by a vote the Hospital Surgeons, with Dr. Bacon, were requested to test its powers, and Dr. H. J. Bigelow was requested to make a subsequent report upon the subject to the Society. As some time must elapse before such a report can be made, Dr. Bigelow has, at our request, furnished us the following statement of his experience of its powers up to the present time.—*Boston Medical and Surgical Journal.*

*Messrs. Editors*:—In reply to your request for information concerning the "kerosolene," and although the evidence is incomplete, I see no impropriety in my furnishing you with such observations as I have been able to make since its introduction to the Medical Society last evening, by Mr. Merrill, Dr. Dickinson and Dr. Bowditch, as an untried agent of suspected anæsthetic properties, which had accidentally affected a man sent in to clean a cistern at the kerosene works, and which had been afterward tried on flies and mice.

This fluid presents remarkable properties. It is tasteless as water, volatile and inflammable as ether, though burning with a dense white light; of a faint chloroform odor, which, as it evaporates, changes to that of coal tar, and then disappears absolutely and altogether; so that a handkerchief saturated with the fluid has, at the end of a few minutes, when dry, no odor at all, nor has the room or atmosphere where it has been used, any trace of its presence. Both ether and chloroform leave, in different degrees, a persistent, *fade* and stale aroma after evaporation, as is well known. They are also far less agreeable to inhale than this new agent, which has thus an obvious advantage over either of them.

A few whiffs were sufficient assurance of its efficacy as an anæsthetic, which, with its other qualities, as I ventured to remark, would place the kerosolene beyond any known anæsthetic, provided its use was not followed by headache, vertigo or other unpleasant symptoms, and provided it should prove as free from danger as ether.

Subsequently, I inhaled the new vapor, which Dr. Hodges at my request administered. Complete insensibility supervened, lasting several minutes, with some diminution of the volume of the pulse. Its effect was wholly agreeable, leaving neither headache nor nausea, nor bad taste.

I have this morning administered it to three surgical patients. The first, a girl of 19, presenting some hysteric tendencies, having thrust some twenty needles in her leg, was wholly insensible during the extraction of four of those which remained. Yet there was more cough than I had expected from the wholly unirritating odor of the vapor, more muscular rigor than usual in favorable anæsthesia, and more intermittence of the pulse.

In a second patient, to whom it was given preparatory to an operation upon the face, insensibility was equally complete. But this woman did not take it kindly, and its complete effect was attended by so feeble and intermittent a pulse as to lead me to desist until she had recovered. A second attempt reproduced, with anæsthesia, the feeble and intermittent pulse, and I again desisted. Upon her recovery, I gave her common ether vapor, which she afterward said was less agreeable, but which was followed by complete insensibility, the pulse beating steadily and full, at 76. Though this patient, perhaps, succumbed more readily to a third anæsthesia, there seemed to be in the two first trials a certain degree of purple color and asphyxia, with its attendant spasm, which I have elsewhere described as an occasional and disagreeable symptom of attempted anæsthesia. To guard against this asphyxia, which might possibly have resulted from the folded towel, upon which I habitually administer ether, I tried in the next case an open sponge. The subject required a considerable incision for a mammary abscess, and was a patient of Dr. H. G. Clark, with whose assent I tried the kerosolene. In spite of the open sponge, the symptoms of asphyxia again appeared, suggesting to Dr. Clark, before operating, their resemblance to those resulting from charcoal gas. The color was livid, and the rigidity marked. In each of those cases, the quantity used was from one to two ounces.

In conclusion, it may be remarked of these three cases, that they are insufficient for satisfactory demonstration, and that their common and unfavorable symptoms may well have been but a coincidence ; yet they suggest some caution in the use of the kerosolene vapor. It is probably more potent than that of ether, requires a free admixture of air, and may produce upon the system some impression or influence, other than that of the mere intoxication attendant upon the use of ether. In awaiting further evidence, it may be considered established that kerosolene is an anæsthetic of undoubted efficiency, and that it possesses certain remarkable and attractive properties peculiar to itself.

*Boston, July 9th,* 1861. H. J. Bigelow, M. D.

